# Risk factors for infection following ingrowing toenail surgery: a retrospective cohort study

**DOI:** 10.1186/s13047-020-00414-y

**Published:** 2020-07-29

**Authors:** Alexander J. Terrill, Katie J. Green, Angelo Salerno, Paul A. Butterworth

**Affiliations:** 1 School of Health and Human Sciences, Southern Cross University, Bilinga, 4225 Australia; 2 Footmed Foot & Ankle Clinics, Adelaide, 5000 Australia

**Keywords:** Podiatry, Ingrowing toenail, Nail surgery, Infection, Onychocryptosis

## Abstract

**Background:**

Ingrowing toenails are a common and painful condition often requiring surgical management. Practitioners who perform surgery on ingrowing toenails include orthopaedic surgeons, general practitioners, podiatrists and podiatric surgeons. There has been limited investigation into the specific surgical approaches used by Australian podiatric surgeons for ingrowing toenails, or the associated infection rates for these procedures. The aim of this study was to assess the frequency and type of ingrowing toenail surgery performed by podiatric surgeons, and identify risk factors for post-operative infection.

**Methods:**

Data was entered into the Australian College Podiatric Surgeons (ACPS) National Audit Tool for all patients who underwent foot and ankle surgery performed by podiatric surgeons in Australia between January 2014 and December 2017. Infection within the first 30 days following surgery was recorded according to the ACPS national audit descriptors. Infection rates, risk ratios (RR) and 95% Confidence Intervals (CI) were calculated to determine postoperative infection risk.

**Results:**

Of 7682 records, 1831 reported 2712 diagnoses of ingrowing nails. Patients with a diagnosis of ingrowing toenails were younger, less likely to have systemic disease, and a lower proportion were female compared to those without ingrowing toenails. Furthermore, they were more likely to be diagnosed with a post-operative infection than those without ingrowing toenails (RR = 2.72; CI = 2.00–3.69; *P* < 0.01). Univariate risk factors for post-operative infection following ingrowing toenail surgery include age greater than 60 years (RR = 3.16; CI = 1.53–6.51; *P* < 0.01), surgery performed in an office setting (RR = 1.77; CI = 1.05–2.98; *P* = 0.04), and radical excision of toenail bed procedure (RR = 2.35; CI = 1.08–5.01; *P* = 0.04). Patients that underwent radical excision or office based procedures were on average older, and more likely to have systemic disease. Further, radical excision procedures were more likely to be performed in office base settings.

**Conclusions:**

Ingrowing toenail surgery carries a greater risk of postoperative infection than other procedures performed by podiatric surgeons. Radical excision of toenail bed was associated with higher postoperative infection rates compared to other ingrowing toenail procedures. Procedures performed in an office setting carry a higher risk of infection. Further research into these associations is recommended.

## Background

Ingrowing toenails may cause pain and a loss of function, and lead to patients seeking treatment from a health practitioner [1]. The prevalence of ingrowing toenails has been measured as 0.46% in a large population study in Korea [2] and 2.45% in the United States [3]. Bennet et al. [4] reported 6.7% of conditions presenting to podiatric surgeons between July 1995 and June 1996 were ingrowing toenails. In the 2017 ACPS audit report [5] this increased to 28.68%. While the increase could be explained by differences in the methodologies between these reports, both highlight that management of the condition constitutes a significant proportion of podiatric surgeons’ workload.

Contributing factors in the development of ingrowing toenails include nail cutting technique, nail shape (curvature), mechanical forces (either from ground reaction forces during gait, compressive forces from footwear, or structural malposition of the digits), hyperhidrosis, injury, and obesity [6]. Because the nail often causes a break in the skin there is potential for pre-existing wound contamination.

A recent systematic review noted that the rates of surgical site infection following any type of foot surgery has been reported as between 0 to 9.4%, and that infection rate is lower where antibiotic prophylaxis is used [7]. A clinical trial in 2007 by Bos et al. [8] looking specifically at infection rates in partial nail avulsion procedures with phenolisation and excisional matrixectomies found no significant difference in the postoperative infection rates between the two approaches. Furthermore, this trial also determined that local administration of antibiotics did not reduce the risk of postoperative infection in either of the two surgical approaches. A retrospective audit of 80 patients receiving a partial or total nail avulsion by Modha et al. in 2016 [9] found an overall postoperative infection rate of 3%.

In Australia, podiatric surgeons are registered specialist podiatrists, with an expanded scope of practice. Podiatric surgeons perform a range of procedures of the foot and ankle, including procedures for digital deformities, nerve entrapments, and ingrowing toenails [4]. The aim of this study was to assess the type of ingrowing toenail surgery performed by podiatric surgeons, and to identify risk factors for infection.

## Methodology

### Data collection

Data was collected by 23 podiatric surgeons using the Australasian College of Podiatric Surgeons’ (ACPS) National Audit tool. Patients were not selected for this study. Rather, their data is collected as a routine component of practice and ongoing audit activity. This tool was developed by an international panel of experts to assess outcomes of foot and ankle surgery. The design, purpose, and method through which surgeons enter data into this tool has been previously described [8, 10–12].

Diagnosis of conditions was recorded by surgeons using the International Classification of Disease, Version 10; Australian Modification coding system (ICD10-AM). For each surgery, a primary diagnosis and up to 30 secondary diagnoses could be recorded. Procedures were recorded using the International classification of Disease, Version 10; Australian Classification of Health Intervention coding system (ICD10-ACHI) which is based on the Australian Medicare Benefits Schedule (MBS). Up to 27 procedures could be recorded for each surgery.

Postoperative infection within 30 days of surgery was recorded according to ACPS Surgical Audit Guide to Data Collection. The ACPS audit tool defines surgical site infection according to the criteria set by the Australian Council on Health Care Standards clinical indicators (version 3.1, 2012). Table 1 summarises the ACPS definition for surgical site infection. Infections were further described as either superficial or deep, and described by the location and the nature of treatment required (i.e. outpatient or admitted). Diagnosis could be made using clinical or microbiological criteria. Infections following surgery were entered into the ACPS Surgical Audit Tool as part of compliance with national audit.

**Table 1:** Criteria for surgical site infection classification, reproduced from the ACPS Surgical Audit Tool guidelines, based upon the Australian Council on Health Care Standards clinical indicators (version 3.1, 2012)

Superficial incisional	Deep incisional
Infection involves only skin and subcutaneous tissue of this incision **AND** Occurs within 30 days after the operative procedure **AND** Exhibits **at least one** of the following from the superficial incision: 1. Purulent discharge (NOT stitch abscess). 2. Organisms isolated from an aseptically collected culture of fluid or tissue. Note: a positive wound swab (in contrast to wound aspirate) without other significant evidence of infection is not adequate for diagnosis of infection. 3. Displays at the site of incision any of the following signs and symptoms of infection: • Pain or tenderness • Localised swelling • Redness or heat **AND** the incision is deliberately explored by the Surgeon resulting in a positive wound culture. Note: A culture-negative finding does not meet this criterion unless the patient was on antibiotics immediately prior to diagnosis. 4. Diagnosis or antimicrobial treatment of superficial incisional infection by the operating Surgeon or Registrar.	Infection involves deep soft tissues (e.g. fascial and muscle layers) AND/OR organs/spaces opened or manipulated during an operation **AND** Occurs within 30 days after the operative procedure if implant not present OR within 1 year if implant insitu **AND** Exhibits either 1 and/or 2: 1. Purulent drainage from deep soft tissue or drain that is placed through a stab wound into the organ/space. 2. Spontaneous dehiscence at the incision site or the wound is deliberately explored by a surgeon with the patient showing evidence of **one or more** of the following signs or symptoms: • Fever 38 °C, localised pain or tenderness with culture positive specimen. A culture-negative finding does not meet this criterion unless the patient was on antibiotics immediately prior to the wound being explored and/or the culture being taken; • Organisms isolated from aseptically obtained culture of fluid or tissue obtained from an organ/space; • An abscess or other evidence of infection involving a deep/organ space is found on direct examination, during re-operation, or by histopathologic or radiologic examination; or • Diagnosis of, or antimicrobial treatment of a deep incisional or organ/space surgical site infection by the operating Surgeon or Registrar.

The American Society of Anaesthesiologists (ASA) score was recorded by surgeons according to previously published methods [12]. The ASA score provides an assessment of patients’ physical status prior to surgery [13]. For the purposes for this analysis, we considered a person with an ASA status of one to have no systemic disease, and a person with an ASA of two or greater to have systemic disease.

Ethical approval was obtained from the ACPS Research Committee to access the ACPS de-identified audit database and was also provided by the Southern Cross University Human Research Ethics Committee (ECN-15-337).

### Analysis

Data recorded by podiatric surgeons between January 2014 and December 2017 were included in the study. Only records which included a valid date of birth, date of surgery, ASA status, and at least one diagnosis and procedure were included in subsequent analysis. No other inclusion or exclusion criteria were applied to the records. For the purpose of this analysis, diagnosis of an ingrowing nail was made using the ICD10-AM code L60.0. Diagnosis of ingrowing toenail was considered as either present or absent regardless of the number of nail edges, digits, or feet that were affected. To calculate infection rates and risk ratios, infection was considered as either present or absent, without considering depth or location of infection.

Demographic and infection risk of patients with ingrowing nails were compared to those without ingrowing nails. Infection rates and univariate risk factors for infection were identified for patients who were exclusively diagnosed with ingrowing nails. Due to the low number of infections observed, we were unable to perform multivariate regression analysis. To identify where potential confounding factors existed, demographic factors were compared for significant risk factors.

Clopper and Pearson exact method was used to calculate 95% confidence intervals for infection rates. Fisher’s exact test (two-sided) and T-Test analyses were undertaken to assess the differences in demographics and infection rate. Statistical analyses were performed using IBM SPSS Statistics (version 25, IBM Corp., Armonk, NY). The level of statistical significance was taken as *p* < 0.05 (5%).

## Results

A total of 8819 surgeries were reported by 23 podiatric surgeons. In the 7682 records which provided complete information, 12,499 diagnoses were identified, of which 2712 were ingrowing toenails, representing 21.7% of all diagnoses. A total of 1831 records (23.8%) reported one or more ingrowing toenails as a diagnosis. Of these, ingrowing toenails were reported as the primary diagnosis in 1661 (21.6%) records, and were reported as the only diagnosis in 1561 (20.3%) records.

### Demographics of patients with ingrowing toenails

Demographics of patients with and without ingrowing toenails are described in Table 2. Patients with ingrowing toenails were on average younger and less likely to have systemic disease than those without. A lower proportion were female compared to those without ingrowing toenails. The surgical procedure was more likely to have been performed in an office-based setting. Patients who had ingrowing toenail surgery were more likely to be diagnosed with a post-operative infection than those without any ingrowing nails.

**Table 2:** Demographics of people undergoing procedures with and without ingrowing nails

	No ingrowing toenail *n* = 5851	Ingrowing toenail(s) *n* = 1831	*P* value
Mean Age (SD)	52.4 (17.5)	39.3 (22.8)	< 0.01* ++
Sex – Female (%)	4539 (77.6%)	1045 (57.1%)	< 0.01* +
ASA 1 (%)	3642 (62.2%)	1290 (70.5%)	< 0.01* +
Office based surgical Facility (%)	862 (14.7%)	875 (47.8%)	< 0.01* +
Total Infections	93	77	
Infection Rate (95% CI)	1.6% (1.3 to 1.9)	4.2% (3.3 to 5.2)	
Infection – Relative Risk	1	2.72 (2.00–3.69)	< 0.01* +

* Statistically significant at *p* < 0.05

+ *P* value calculated using Fisher’s exact test (two-sided)

++ *P* Value calculated using Independent samples T Test, Equal variances not assumed

ASA 1 = no systemic disease.

**Fig. 1:**
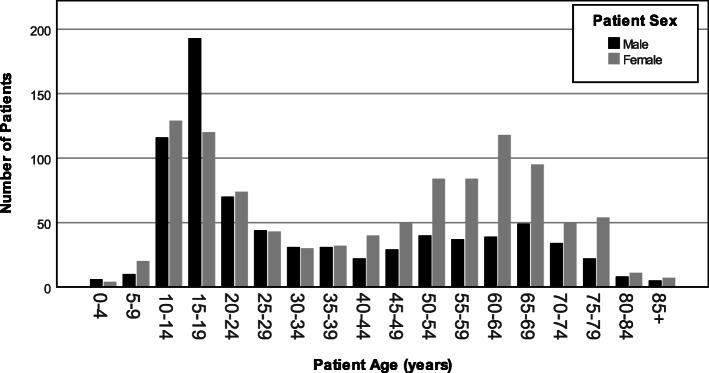
Age and sex of patients diagnosed with ingrowing nail(s)

There was a bimodal age distribution for patients with ingrowing toenails, with peak diagnoses reported between ages of 10 to 19, and 60 to 64 (Fig. 1). Males were represented in greater numbers in patients with ingrowing nails under 40 years of age, and females were represented in greater numbers in patients over 40 years of age.

There were 32 other diagnoses identified in the 1831 patients who were diagnosed with ingrowing nails. The most common diagnoses were osteophyte (exostosis) (154 cases, 8.4% of patients with ingrowing nails), hammer toe deformities (68, 3.7%), and hallux valgus (28, 1.5%).

There were 61 different types of surgical procedure performed on patients with ingrowing toenails. Patients could have received multiple procedures. The most common being wedge resection (performed for 54.3% of patients), partial resection (33.6%), radical excision of the nail bed (7.4%), ostectomy of toe (5.8%), excision of exostosis (4.2%), and total removal of nail (3.9%). The most common procedures for management of ingrowing nails are described in Table 3.

**Table 3:** Descriptions of surgical procedures for management of ingrowing toenail [14]

Item code	Procedure name	Procedure description	Example
47915	Wedge Resection	Wedge resection with removal of segment of nail, ungual fold and portion of the nail bed	Winograd matrixectomy
47916	Partial Resection	Partial resection of nail, with destruction of nail matrix by phenolisation, electrocautery, laser, sodium hydroxide or acid but not including excision of nail bed	Partial nail avulsion with phenolisation
47918	Radical Excision	Radical excision of nailbed	Terminal syme
47906	Total Removal	Removal of digital nail of toe	Total nail avulsion

### Risk factors for infection following ingrowing toenail surgery

There were 1561 patients who were diagnosed with ingrowing toenails only, of whom, 65 (4.2%; CI = 3.2–5.3%) were diagnosed with infection following the procedure. Of these patients, 60 were diagnosed with superficial forefoot infection, two were diagnosed with deep forefoot infection, one with superficial midfoot infection, and two with superficial rearfoot infection. No infections necessitated hospital admission for treatment. Infection rates and risk factors for infection in patients diagnosed with ingrowing toenails is described in Table 4. Risk of infection increases with increasing age, and is higher for patients who undergo radical excision of the nail bed, or, who have a procedure in an office setting.

**Table 4:** Risk factors for infection following surgery in patients with ingrowing nails only

	Infections / n	Infection rate (95% CI)	Risk ratio (95% CI)	*P* value+
**Age**				
< 18	11 / 475	2.3% (1.2–4.1)	1	
18–59	29 / 727	4.0% (2.7–5.7)	1.75 (0.87–3.54)	0.14
≥ 60	25 / 359	7.0% (4.6–10.1)	3.16 (1.53–6.51)	< 0.01 *
**Sex**				
Male	32 / 737	4.3% (3.0–6.1)	1	
Female	33 / 824	4.0% (2.8–5.6)	0.92 (0.56–1.51)	0.80
**ASA**				
1	44 / 1163	3.8% (2.8–5.0)	1	
≥ 2	21 / 398	5.3% (3.3–8.0)	1.42 (0.83–2.41)	0.19
**Surgery Facility**				
Hospital/Surgicentre	22 / 732	3.0% (1.9–4.5)	1	
Office	43 / 829	5.2% (3.8–6.9)	1.77 (1.05–2.98)	0.04 *
**Procedure**				
Wedge Resection	31 / 743	4.2% (2.9–5.9)	1	
Partial Resection	22 / 578	3.8% (2.4–5.7)	0.91 (0.52–1.59)	0.78
Radical Excision	9 / 97	9.3% (4.3–16.9)	2.35 (1.08–5.01)	0.04 *
Total Removal	2 / 46	4.3% (0.5–14.8)	1.04 (0.24–4.50)	1.00
Multiple/other procedures	1 / 97	1.0% (0.0–5.6)	0.24 (0.03–1.77)	0.16

+ *P* value calculated using Fisher’s exact test (2-sided)

* Statistically significant at *p* < 0.05

### Association between type of procedure, surgical facility, and patient demographics

While infection risk is greater following procedures performed in an office setting, those undergoing the procedure in an office setting are more likely to have systemic disease, and have a higher average age (Table 5).

**Table 5:** Demographics of patients undergoing procedure according to type of surgical facility

	Hospital/Surgicentre *n* = 732	Office *n* = 829	*P* value
Mean Age (SD)	32.4 (21.5)	40.4 (22.7)	< 0.01* ++
Sex – Female (%)	381 (52.0%)	443 (53.4%)	0.61 +
ASA 1 (%)	565 (77.2%)	598 (72.1%)	0.02* +

* Statistically significant at *p* < 0.05

+ *P* value calculated using Fisher’s Exact test (two-sided)

++ *P* Value calculated using Independent samples T Test, Equal variances not assumed

ASA 1 = no systemic disease.

Those who underwent a wedge, partial, or total resection of the nail were on average younger and less likely to have systemic disease than those that underwent a radical excision of the nail bed (Table 6). Additionally, those who underwent radical excision were more likely to have the procedure performed in an office setting.

**Table 6:** Demographics of patients undergoing procedure according to type of procedure

	Radical excision *n* = 97	Partial, wedge, or total nail resection ^a^ *n* = 1367	*P* value
Mean Age (SD)	54.8 (20.8)	34.6 (22.0)	< 0.01* ++
Sex – Female (%)	55 (56.7%)	703 (51.4%)	0.35 +
ASA 1 (%)	50 (51.5%)	1047 (76.6%)	< 0.01* +
Office Based Facility	76 (78.4%)	710 (51.9%)	< 0.01* +

^a^*Other/Multiple procedures were excluded from this grouping.*

* Statistically significant at *p* < 0.05

+ *P* value calculated using Fisher’s exact test (two-sided)

++ *P* Value calculated using Independent samples T Test, Equal variances not assumed

ASA 1 = no systemic disease.

Those who underwent a wedge resection in an office-based setting were at a greater risk of postoperative infection compared to those who had the same procedure in hospital or surgicentre (Table 7). There was no such risk increase for those patients who had a partial resection. The number of infections in those who had a radical excision of the nail bed or total removal were too low to be included in this analysis.

**Table 7:** Infection rates depending on procedure and location

	Infection/n	Infection rate (95% CI)	Risk ratio (95% CI)	*P* value
**Wedge Resection**				
Hospital/Surgicentre	14 / 480	2.9% (1.6–4.8)	1	
Office	17 / 263	6.5% (3.8–10.1)	2.30 (1.12–4.75)	0.03* +
**Partial resection**				
Hospital/Surgicentre	6 / 171	3.5% (1.3–7.5)	1	
Office	16 / 407	3.9% (2.3–6.3)	1.13 (0.43–2.93)	1.00 +

+ *P* value calculated using Fisher’s exact test (two-sided)

* Statistically significant at *p* < 0.05

## Discussion

In a previous clinical audit of podiatric surgeons, Bennett [4] examined 786 patient files between July 1995 and June 1996. Bennett [4] found 106 of 1575 (6.7%) reported procedures were for ingrowing nails. In this current study, we have found the frequency of presentation of ingrowing nails to podiatric surgeons to be higher (21.7% or all presenting conditions). These differences may be attributed to the differences in data collection. The current study reviewed data collected using the ACPS audit tool. Data is entered into this system in real time by Podiatric surgeons. Bennett [4] reported data collected by a researcher retrospectively reviewing clinical records. This collection method may therefore have not identified all diagnoses of ingrown toenails within these records. Moreover, podiatric surgery has progressed since Bennett [4] undertook the study, establishing itself as a mainstream surgical profession, leading to increased referral pathways and service access. For minor procedures that may have otherwise been performed by a general practitioner, or may have resulted in a long wait list time in the public system, podiatric surgeons provide an alternate pathway.

Similar to a recent study [2], we have identified that ingrowing nails have a bimodal age distribution, with peaks in teenagers and people aged 40–50 years. Males were represented in greater numbers under the age of 40 and females were represented in greater numbers over the age of 40. The peak in prevalence of ingrowing toenails in teenagers could be attributed to greater physical activity in this age group, as an association between high levels of physical activity and ingrowing toenails in young males has been previously been demonstrated [15]. Poorer nail cutting practices and hyperhidrosis may also account for the higher rates of ingrowing toenails seen in this population [6]. The peak seen in the 40–50 year age group and the greater representation of women in this group could be accounted for by the increased prevalence of foot deformities (such as hallux abducto valgus) in older populations and females [16]. Such foot deformities have been described as a risk factor for the development of ingrowing toenails [2]. Older populations, in particular older females, are also more likely to wear ill-fitting shoes [17] that create compression forces on the toenail and play a role in the development of ingrowing toenails [6].

As described by Levy [3] and Cho [2], females represent a greater proportion of people affected by ingrowing nails, although ingrowing nails are also prevalent in younger males. However, Menz et al. [18] found that more nail procedures were performed for males than females by private surgeons. Our results showed that women were more likely to have surgery for an ingrown toenail than men, accounting for 57% of patients undergoing surgery for ingrowing toenails. In addition to the presence of foot deformity and poor footwear, the greater number of women undergoing nail surgery could be attributed to the fact men may be less likely to access health care [19].

In this current study, we identified that the rate of infection is higher for people undergoing procedures for ingrowing nails, than those undergoing procedures for other foot pathology. However, the rate of infection is lower than that reported in a similar study exploring postoperative infection after excisional toenail matrixectomy [20]. The elevated rate of infection may be due to the presence of pre-existing wound contamination or infection.

Alternatively, infection rates may be elevated due to the perceived ‘less-serious’ nature of nail procedures compared to surgeries of bone and joint. This perception could potentially reduce the likelihood of antibiotic prophylaxis, or lead to procedures being performed in office-based rather than hospital facilities where intravenous antibiotic prophylaxis is available. Further, infection rates may be elevated because of reduced patient adherence to postoperative instructions. Given the high number of procedures performed by podiatric surgeons in an office setting, and the increased infection rate in this group, podiatric surgeons should consider the procedure location, and antibiotic prophylaxis, in their decision-making process.

The use of antibiotics in in the surgical management of ingrowing nails is unclear. Córdoba-Fernández et al. [21] recommended the use of prophylactic antibiotics only in infective ingrowing toenails. A Cochrane review revealed that the use of antibiotics has not been shown to result in a difference in infection rates [22]. Using a similar dataset, a prospective cohort study by Butterworth et al. [11] showed that the use of perioperative antibiotics in foot and ankle surgery may reduce the risk of infection. However, Butterworth et al. [11] did not separate their study population into specific procedure groups. Consequently, whether antibiotic prophylaxis reduces infection rates following ingrowing toenail surgery may be unclear. However, considering that many ingrowing toenail procedures are performed by podiatric surgeons under local anaesthetic, in office settings, where perioperative antibiotics may not be available, changes to practice should be considered to reduce infection rates in this population. Given that peri-operative antibiotic prophylaxis is standard procedure in hospitals, patients undergoing sharp excisional ingrowing nail surgery should be admitted for these procedures.

Our current study showed that the choice of procedure for management of ingrowing nails is associated with patient age and ASA status. Patients who underwent radical excision were on average older, and more likely to have systemic disease than those who underwent wedge or partial resection. This could be due to increased concern regarding healing times following chemical ablation in the older patient with systematic disease, or concern regarding long term aesthetics for the younger person. Alternatively, wedge and partial excisions may commonly be utilised as an earlier line of treatment. Ingrowing toenails of greater severity or associated with gross deformation or disease of the nail, or ingrowing nails recalcitrant to treatment, may require radical nail bed excision. Procedure choice may be dependent on the presence of peri-ungual soft tissue variation seen around the nail fold, such as ungelabia and onychomatrixoma. Procedure choice may also be due to associated bone pathology such as subungual exostoses, which is being addressed concomitantly. Furthermore, a surgeon may choose an excisional matrixectomy over a chemical procedure to aid direct visualisation of target tissue when revision surgery is being performed in the case of primary procedural failure.

In the current study, a higher infection rate has been associated with radical excision procedures. The nail and nail folds have been identified as the most heavily contaminated area of the foot and ankle and are difficult to decontaminate [23]. Radical nail bed excision potentially disseminates debris from the nail folds, contaminating subcutaneous tissue and periosteum overlying the distal phalanx. Radical bed excision may also be a procedure of choice for more severe ingrowing toenails with active or unresolved paronychia, hyper-granulation tissue and bone deformity. The higher infection rate may be attributed to an unresolved infection at the time of surgery. However, patients undergoing radical excision were more likely to be older, have systemic disease, and have the procedure performed in an office setting. Consideration should, therefore, be given to admitting these patients to a day surgery facility, especially those with systemic disease who are aged over 60.

This study has identified that some procedures (wedge resection) for ingrowing nails performed in an office setting are associated with a higher rate of infection. This could be due to the greater emphasis on the design and maintenance of hospital and day surgery facilities for pathological microorganism control. However, those patients undergoing procedures in office settings were also older, and more likely to have systemic disease. A question raised in this study is why are older patients with systemic disease more likely to have a procedure performed outside a hospital setting? It could be convenience, cost (including lack of Medicare and insurance funding), or a combination of these and other factors. Further research is required to answer this question. Of interest to the podiatric profession is that no increase in infection risk was identified for partial resection (with phenol) in an office setting. This result is likely due to partial resections being less invasive, not involving skin incisions, or bone. Moreover, phenol has been shown to provide anti-bacterial activity against both gram positive and gram negative bacteria [24].

In this current study, we have shown that certain population groups have a higher reported rate of infection. However, due to the low rate of infections, we were unable to perform multivariate regression analysis to control for other factors. For example, whilst the rate of infection is higher in patients who underwent radical excision of nail bed, the average age of a patient who had this procedure was also higher, and they were also more likely to have systemic disease.

There are limitations in this study which could have resulted in misreporting of infection rates. Infection could have been identified by the surgeons based on clinical manifestations only, and did not require microbiological confirmation in all cases. Further, it is possible that some patients would present to other members of their health care team (for example general practitioner) in the first instance of a minor post-surgical infection. Also, it is possible that infection rates were affected by those patients with a pre-existing infection. Further, no information was available regarding the use of antibiotics.

The follow up period for this study was 30 days and did not consider whether the procedure resulted in permanent resolution of the patient’s condition. It is possible that patients who present to podiatric surgeons have had previous ingrowing toenail surgery, or more complex health concerns than those patients who present to a non-specialist podiatrist, therefore, these results should be interpreted with caution outside of these settings. However, a key strength of this research design is that it measures real-world practice.

## Conclusion

Ingrowing toenail surgery is associated with a greater risk of postoperative infection than other procedures performed by podiatric surgeons. Radical excision of toenail bed was associated with higher postoperative infection rates compared to other ingrowing toenail procedures. Some procedures performed in an office setting carry a higher risk of infection. Further research into the associations identified in this study is recommended. Specifically, controlled and prospective studies that investigate outcomes of ingrown toenail procedures would demonstrate causative links with important variables. Further exploration of the factors which contribute to decision making by podiatric surgeons regarding the type or settings of surgery would be of benefit. Finally, a comparison of microbiology confirmed versus clinically confirmed diagnosis of infection may help with reporting accurate infection rates.

## Data Availability

The data that support the findings of this study are not publicly available. Data are however available from the authors upon reasonable request and with permission of the Australian College of Podiatric Surgeons.

## Abbreviations

ACPS: Australasian College of Podiatric Surgeons
ASA: American Society of Anaesthesiologists
ICD10-ACHI: International classification of Disease, Version 10; Australian Classification of Health Intervention
ICD10-AM: International Classification of Disease, Version 10; Australian Modification
MBS: Medicare Benefits Schedule
RR: Risk Ratio
CI: Confidence Interval
